# National and subnational size estimation of female sex workers in Ghana 2020: Comparing 3-source capture-recapture with other approaches

**DOI:** 10.1371/journal.pone.0256949

**Published:** 2021-09-22

**Authors:** Chris Guure, Samuel Dery, Seth Afagbedzi, Waimar Tun, Sharon Stucker Weir, Silas Quaye, Augustine Ankomah, Kwasi Torpey

**Affiliations:** 1 Department of Biostatistics, School of Public Health, University of Ghana, Accra, Ghana; 2 HIV and AIDS Program, Population Council, Washington, DC, United States of America; 3 Department of Epidemiology, Carolina Population Center, University of North Carolina, Chapel Hill, NC, United States of America; 4 Division of Global HIV/TB, Center of Global Health, US Centers for Disease Control and Prevention (CDC), Accra, Ghana; 5 Department of HIV and AIDS, Population Council, Accra, Ghana; 6 Department of Population, Family and Reproductive Health, School of Public Health, University of Ghana, Accra, Ghana; University of Yaounde I, CAMEROON

## Abstract

**Background:**

Key Population size estimation (PSE) is instrumental for HIV/STI preventive, treatment and care services planning, implementation and delivery. The objective was to estimate the overall population of female sex workers (FSW) in all the 16 regions of Ghana using different PSE methods.

**Method:**

Mapping of venues and complete enumeration of seaters was conducted at the formative stage prior to the bio-behavioral survey (BBS). Three PSE methods were used to derive the size estimates of FSW in the 16 regions. These include: Capture-recapture (CRC), service multiplier and three-source capture recapture (3SCRC) methods. The final choice of the estimation method used to estimate the roamer population was 3SCRC. This method was chosen because of its perfect record-linkage–hierarchic combination of three names that minimizes overmatching as well as the addition of an interaction term in the model which corrects for the dependencies in CRC.

**Results:**

The total population size estimate of the female sex workers in the country obtained for roamers using capture re-capture was 41,746 (95% CI: 41,488–41,932). Using the service multiplier, the total population for both the roamers and seaters was 41,153 (95% CI: 37,242–45,984). The 3-source capture re-capture yielded 55,686 roamers FSW (95% CI: 47,686–63,686). The seater population was 4,363 FSW based on census/complete enumeration. The total population size estimate of FSW (seaters and roamers) in Ghana was 60,049 when 3SCRC and census were added. This represents about 0.76% of all estimated adult females aged 15-49yrs in Ghana.

**Conclusion:**

We report population size estimates (PSE) for FSW in Ghana. These estimates are the results of 3SCRC. These findings provide a valid and reliable source of information that should be referenced by government officials and policymakers to plan, implement and provide HIV/STI preventive, treatment, and care services for FSW in Ghana.

## Background

Ghana’s HIV epidemic is described as generalized, with a current estimated adult prevalence of 1.67% [1]. While the prevalence may be relatively low compared to other African countries, results of integrated biological and behavioral surveillance surveys-2015 (BBS) among key populations (KP) estimated an HIV prevalence several times higher than that of the general population [2]. The geographical heterogeneity and high HIV prevalence among KPs underscore the vulnerabilities that have the potential to influence the spread of the epidemic in the country.

Even though the HIV epidemic in Ghana is described as generalized, it is characterized by a significant contribution from transmission among Female Sex Workers (FSW) and their clients, as well as among men who have sex with men (MSM), many of whom also have female sex partners. The Modes of Transmission (MoT) analysis conducted in 2014 in Ghana suggests that 12% of new HIV infections occurring among those aged 15–49 years old were among FSW, MSM, and clients of FSW. The MoT study estimated that an additional 13% of new infections occur among regular female partners of MSM and clients of sex workers. This suggests that MSM and FSWs, together with female partners of MSM and male clients of FSWs, account for about 25% of new HIV infections in Ghana [2]. The 2015 BBS of FSWs in ten regions found an overall HIV prevalence of 6.9% ranging from 2.9% in the Upper East Region to 9.0% in the Ashanti and Greater Accra Regions [2]. The National HIV and AIDS Monitoring and Evaluation Plan (2016–2020) provided for mapping and size estimation of KPs in Ghana every 2–3 years. This is expected to provide the needed information to guide HIV programming for KPs according to the Ghana AIDS Commission-2016. The FSWs PSE will enable appropriate allocation of resources and guide the development and implementation of appropriate interventions specifically targeting them in various geographic locations to maximize program impact.

Population size estimation is a challenge because the KPs are hidden and have fluid identities based on risk behaviors that may change over time. For these reasons, multiple methods should be used to obtain multiple PSE and establish plausible ranges as against single method due to variability [3–6]. In recent times, a number of studies have been carried out using varied methods to estimate the size of key populations. These include multiplier method, Capture-recapture (CRC) [6–11], Respondent-driven sampling (RDS) through successive sampling-population size estimation (SS-PSE) using imputed visibility and three-source capture-recapture (3SCRC) [12–14] and Census and Enumeration [15].

The objective of this study was to estimate the total population of FSW in Ghana using three PSE methods and census/enumeration. The estimation methods were: Capture-recapture (CRC); Service multiplier and three-source capture-recapture (3SCRC). In addition, because there were known probabilities of selection at each stage of the survey sampling process, we were able to compare calculated size estimates with the weighted sample size frequencies estimated by survey methods (SAS/STAT 14.2 User’s Guide, The Surveyfreq Procedure).

## Methods

### Target populations

The operational definition of FSWs was kept the same and aligned with the National HIV and AIDS Strategic Plan 2016–2020 of the Ghana AIDS Commission and the M&E/Surveillance Working Group. An FSW was defined as any female aged 16 years (i.e., the age for consensual sex in Ghana) or older who reported having exchanged sexual acts (e.g., vaginal, anal and/or oral sex) in the last 6 months with someone other than her established partner for something of value (money and material items) that would otherwise not be extended to them by their sexual partners [16].

Two main categories of FSWs were considered in this study, namely **seaters** and **roamers**. **Seaters** were defined as FSWs who operate their sex work at specific defined/fixed and well-known locations such as homes and brothels including hotels, lodges etc. These FSWs have some form of permanent affiliation or connection with these venues. **Roamers** on the other hand are FSWs who are not associated with a specific defined location but who usually move from one location to another to seek clients.

### Ethical considerations

This study received ethical approval from the University of Ghana Noguchi Memorial Institute of Medical Research Institutional Review Board (CPN 083/18-19), the Ghana Health Service Ethical Review Committee (GHS-ERC 002/05/19) and the Population Council Institutional Review Board (Protocol 891), New York, USA. Study participation was voluntary, and respondents were informed that they were free to withdraw from the study at any time during the survey process. Following careful explanation of the survey, study staff gave eligible respondents the consent form to read or, if necessary, had the consent form read to the respondents by a staff member. All respondents signed that they understood and agreed to all the items contained in the consent form before being enrolled in the survey.

### Geographic areas

The survey was conducted in the 16 regions of Ghana: Greater Accra, Ashanti, Western, Western North, Central, Eastern, Volta, Oti, Bono, Ahafo, Bono East, Northern, Savannah, North East, Upper East and Upper West.

### Study design

The study was operationalized and implemented in three phases from August 2019 to June 2020.

### Phase 1: Pre-survey assessment, venue verification and mapping

Several activities were conducted in this phase. They included national and regional stakeholder consultations with civil society organizations (CSOs) with experience of working with FSWs. Given that sex work is illegal in the country, the Ghana Police Service was a key stakeholder. At the regional level, the existing lists of venues where FSWs could be found were reviewed and updated. These lists of existing venues were obtained from the 2015 IBBSS study, program implementers, peer educators and the Ghana Police Service. Furthermore, key informant interviews were also used to identify additional venues. During the key informant interviews, the following questions were asked: What is the name of the venue / spot? Are there additional names for the venues? What is the address or street of this place? Do women who have sex for money visit this place? Do female sex workers go there? Is this a place where female sex workers look for customers on the street? Is this a place where women live and do sex work on site? All the venues that were found or identified were verified and mapped using Geographic Information System (GIS) techniques.

### Phase 2: Population size estimation using capture-recapture methodology (CRC)

In this phase, a comprehensive list of validated and mapped venues was built from Phase 1. Fifteen percent of the venues listed were randomly sampled and visited by trained research assistants who distributed unique objects to FSWs who met the eligibility criteria. The following screening questions were used to determine the eligibility of the respondents: Do you come here often? Do you think men come here to meet women? Sometimes men give money or gifts to women for sex. Has a man other than your main partner given you gifts or money for having sex with him? If yes: in the past 6 months, has any man given you gifts or money for sex? If yes: Are you aged 16 or older? (A person passes the screen if yes to 3, 4 and 5). In the first capture, branded wrist bands were distributed to all eligible FSWs on-site across the country. The number of FSWs who were shown the wrist bands as well as those who accepted them at each venue were recorded. In the second capture which took place the following week at the same venues, hand branded sanitizers were also distributed to the FSWs on-site irrespective of whether or not they were present during the first capture. Again, the numbers of FSWs who were present, eligible, and either received/ accepted or did not accept the hand sanitizers were also recorded. Size estimates of the roamer FSWs at the randomly sampled 15% of venues were calculated and extrapolated to cover a 100% of the FSW population. The extrapolation was done for all of Ghana (each region had a unique wrist band design). The formula used for the population size estimation based on the 15% sample is given below:

### Phase 3: Bio-behavioral survey

The main BBS survey (behavioral and biological) took place in this phase. The sampling strategy was time-location sampling, operationalized by randomly selecting venues from the master list of venues and subsequently sampling peak times as documented during visits to the venue. All women present at the time of the interview were approached and screened to determine eligibility status and to obtain informed consent. Those who were eligible and consented were taken to a nearby private location, interviewed, and tested for specific STIs.

During the survey, each respondent was asked (shown the physical objects) about whether she received the first unique object (wrist bands) as well as second unique object (hand sanitizers). Also, information about those who took part in either one, two or the three (part of the survey) was collected and used to estimate the FSW population size via the three-source capture-recapture methodology.

### Population size based on survey weights

Because the survey sample was a probability sample with known probability of selection for each respondent, a survey weight was calculated for each respondent based on her probability of selection into the survey, taking into account the probability of selection of the venue-time period and the number of available potential respondents at the venue. SAS surveyfreq was used to estimate the weighted frequencies and appropriate confidence intervals, taking into account the survey design. This method was not initially proposed for size estimation but was considered appropriate method for comparison because of the rigorous sampling methods and documentation of sampling probabilities.

### Data collection tools, techniques and training

In the survey, a structured questionnaire was adapted from Global HIV Strategic Information Working Group [17]. Further modifications were made after pretesting. Data were collected on secure tablets and uploaded to servers at University of Ghana using standard protocols to ensure confidentiality.

### Data management

The data manager and the biostatistician employed best practices and standard procedures for data management and processes to achieve the study’s goals and objectives. Data were captured on the field using REDCap. An online version of the study questionnaire was designed for REDCap and uploaded onto a mobile (android and IOS) app that is in sync with the REDCap software; this enabled the research assistants to synchronize data directly to the server. The use of the mobile application reduced the time in data collection and processing and facilitated real-time monitoring of the data collection.

During the field data collection, data quality issues were identified in real-time by 15 trained data monitors and data cleaning and validation occurred simultaneously. All electronic data capturing devices (tablets and laptops) were password protected.

### Data analysis

Mapping and size estimation data were tabulated and analyzed using Excel, Stata Version 16. The techniques behind each of the three methods used, is briefly described in the following sub-sections.

#### Service multiplier

The multiplier method requires at least two aggregate data sources, service data and survey. Service data were obtained from the United States Agency for International Development (USAID) funded Continuum of Care Project and the Global Fund supported West Africa Program to Combat AIDS (WAPCAS) project. These were the only two FSW intervention projects operational in the country at the time of the study. The study obtained data on the number of FSWs reached by peer educators between July 1 and December 31, 2019 by the two projects. The second data source was from the BBS. The proportion of FSWs who reported having been reached by a peer educator from these projects between July 2019 and December 2019 was determined. An estimate was obtained using a formula given as

$$S=\frac{\#of\;FSWs\;reached\;by\;peer\;educators\;from\;the\;projects}{\%FSWs\;reported\;being\;reached\;in\;survey\;}$$

where *S* is the population size.

#### Capture-Recapture (CRC)

The formula for the population size estimation for the capture-recapture approach based on the 15% sample is:

N=MC/R

Where,

N = Estimate of total population size

M = Total number of FSWs who received the wrist bands during the first capture

C = Total number of FSW who received the hand sanitizers during the second capture

R = Number of FSWs captured on the first visit that were then recaptured on the second visit.

#### Three-Source Capture–Recapture (3SCRC)

**Fig 1:**
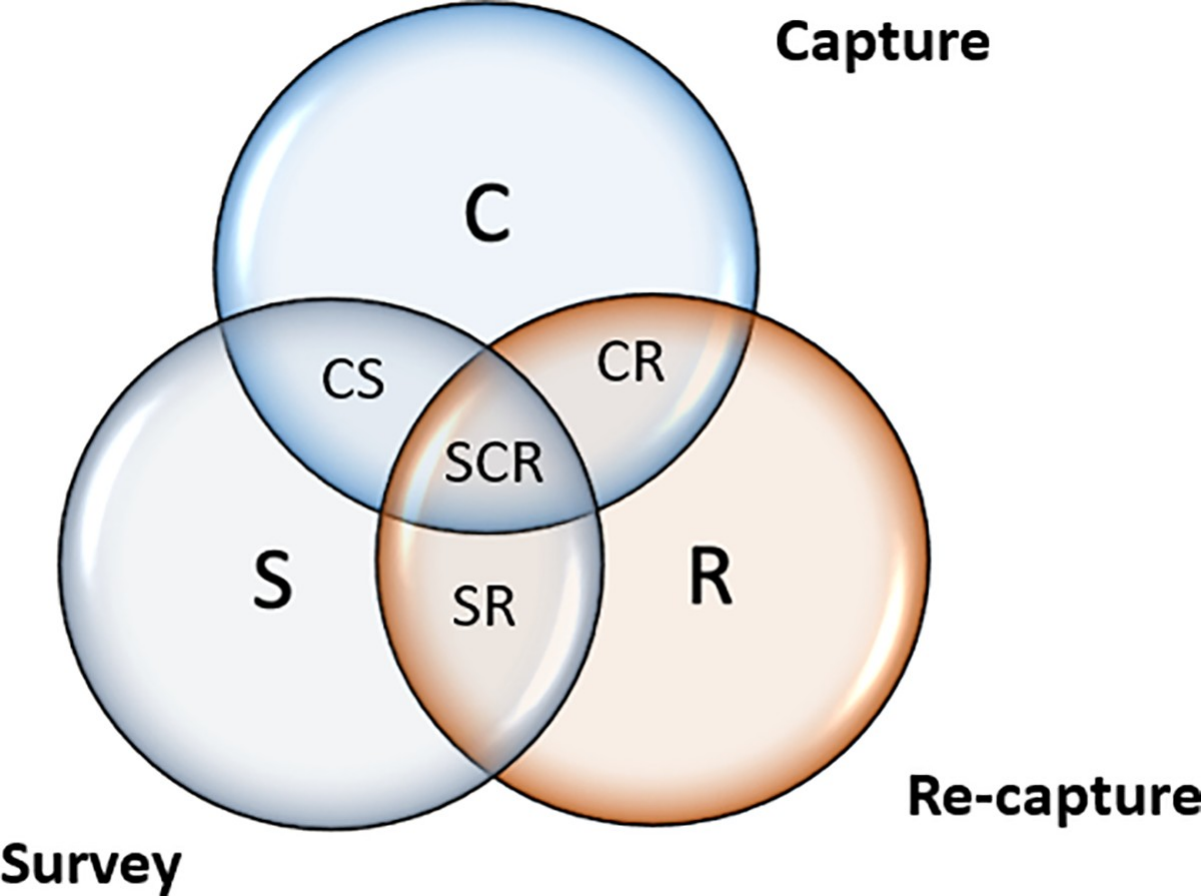
Framework used to obtain population size estimates for roamer FSWs using 3-source capture-recapture approach.

A 3-source capture-recapture approach is methodologically more robust than other PSE approaches. 3SCRC deals with some of the limitations that CRC has, such as dependency between samples. In this approach, information was collected about FSWs who were captured during the first and the second events as well as the survey. The 3SCRC information was used for the following eight (8) models using Fig 1.

1. FSW who were in the first capture only (C)
2. FSW who were in the second capture only (R)
3. FSW who only took part in the survey (S)
4. FSW who were captured in both (C) and (R) but not (S)
5. FSW who were captured in both (C) and (S) but not (R)
6. FSW who were captured in both (R) and (S) but not (C)
7. The number of FSW who were captured in all the three sources (CRS)
8. The number of FSW who were not captured

An assumption of homogeneity for each capture was specified and analysis carried out using a Stata user-written program **(**https://ideas.repec.org/c/boc/bocode/s456859.html**)**.

## Results

### Section 1: Mapping and estimates of the FSW population

**Fig 2:**
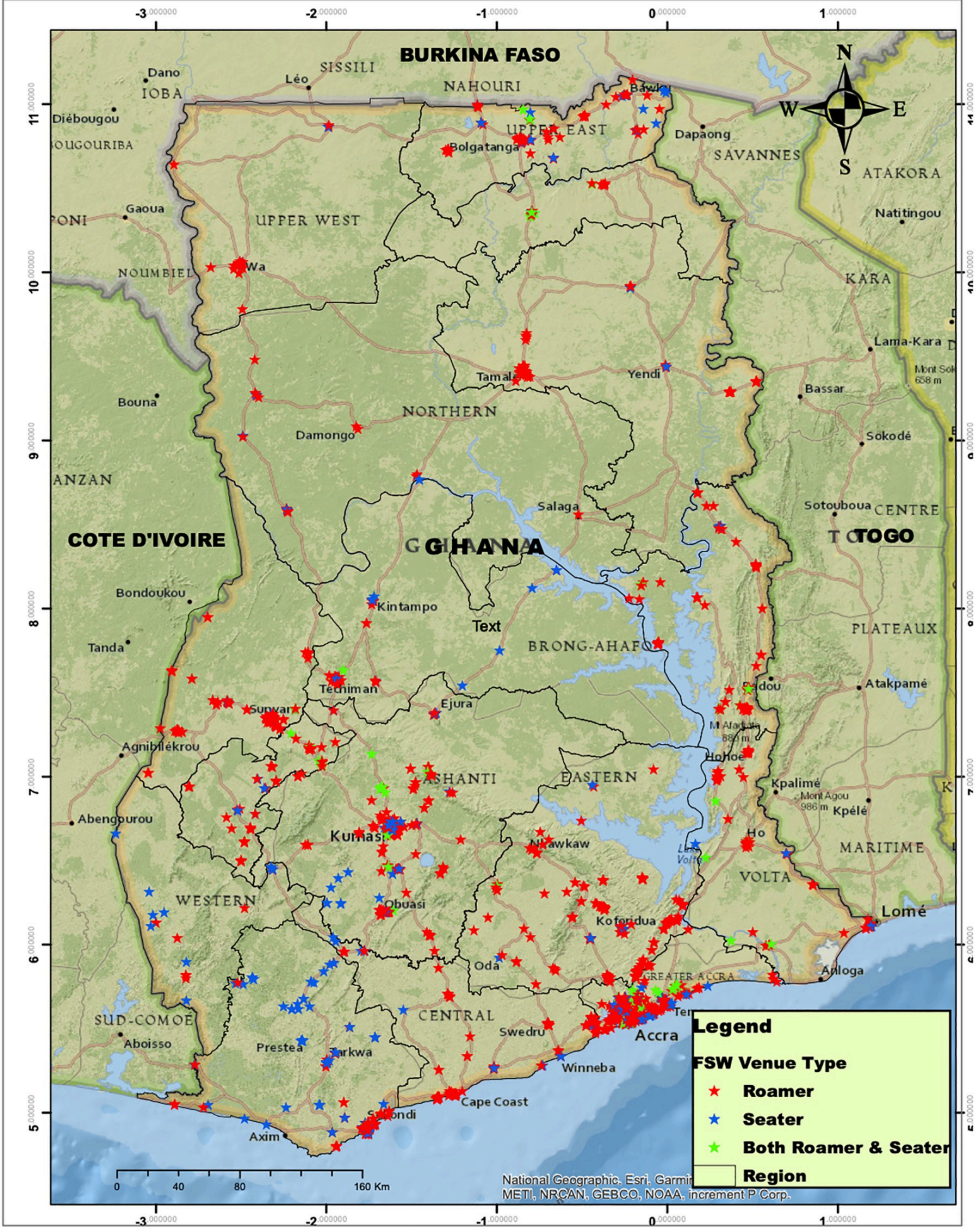
Distribution of FSW venue types among the 16 regions of Ghana, 2020.

**Fig 3:**
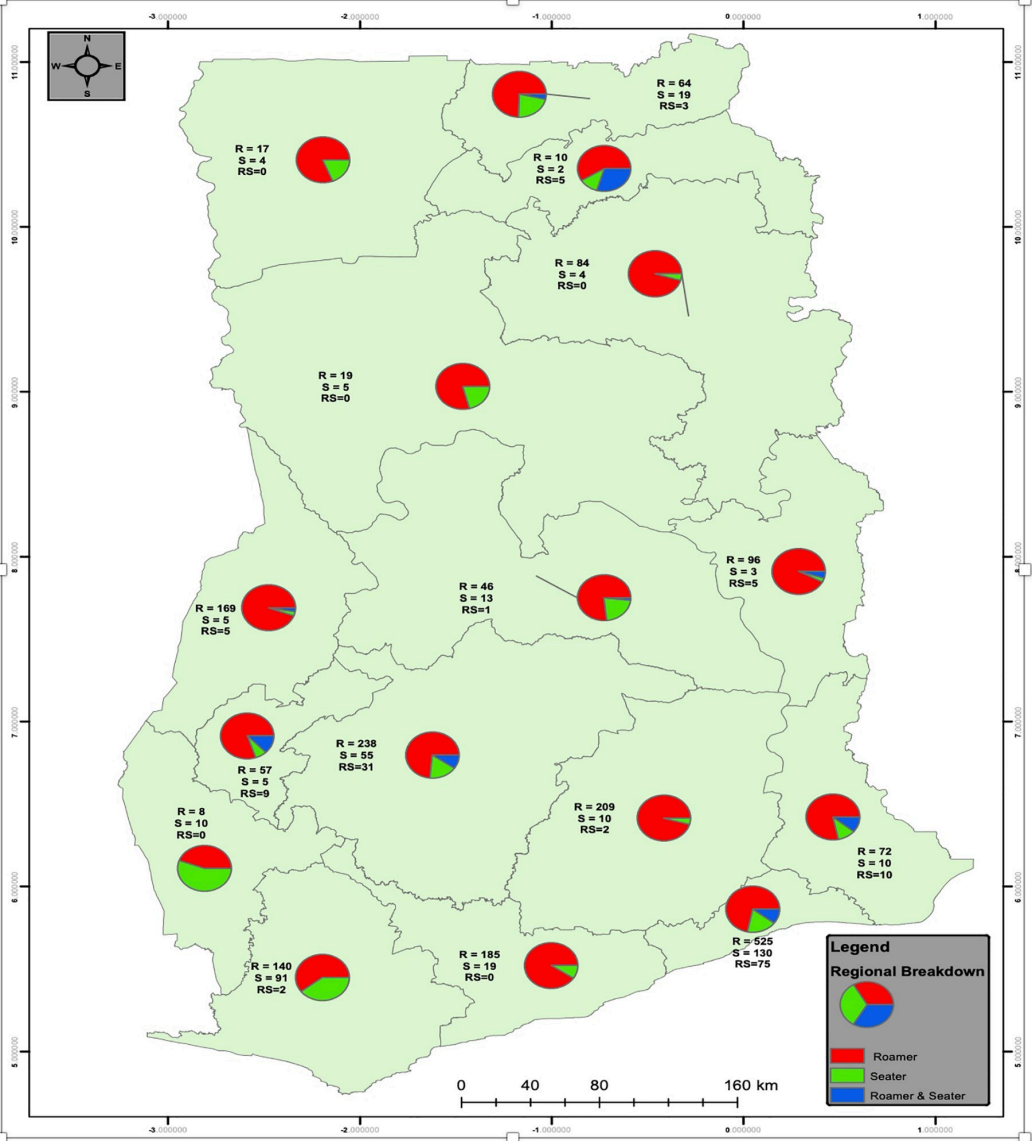
Frequency distribution of venue types among the 16 regions of Ghana, 2020.

The total number of active FSW venues found and mapped nationwide were 2,482 (Figs 2 and 3). Figs 2 and 3 depict the regional distribution of FSW venues. The distribution of the venues by region is provided in Fig 3. The majority of venues belong to the roamer category across all the 16 regions of Ghana. Though all regions have seater venues, the numbers across the country are generally small. Most of these venues are clustered around the regional capitals, especially Greater Accra, Ashanti, Western, Bono and Upper West (Fig 2). The majority of FSW venues (Figs 2 and 3) are located within the Southern and Middle belts of the country. Both North East and Western North have the smallest number of venues.

### Estimation of the roamer population using capture-recapture

In the first capture in the 16 regions, a total of 3,430 roamers participated. The highest number of participants were in Greater Accra region (1,122 representing 33% of the entire country). The least number of captures occurred in the Savannah Region- 10 (0.3%). In the re-capture, there were 3,533 roamers. Out of the number of captures in the second round, 1,964 were part of the first capture, leaving a total of 1,569 new second round captures. This represents about 57% of the first captured participants recaptured. This method yielded a size estimate of 6,164 for the 15% sample of venues, which was extrapolated to a total size estimate of 41,746 (95% CI: 41,488–41,932) FSWs (roamers) in the country, Table 1.

**Table 1:** Population size of roamer FSW using capture-recapture by region in Ghana, 2020.

Region	Est	95% LCI	95% UCI
Western	4,128	4,042	4,214
Central	4,602	4,530	4,672
Greater Accra	13,751	13,626	13,874
Volta	1,045	1,007	1,083
Eastern	3,426	3,378	3,474
Ashanti	6,016	5,904	6,126
Western North	254	220	230
Ahafo	703	666	740
Bono	2,353	2,313	2,391
Bono East	737	716	756
Oti	966	907	1,025
Northern	1,463	1,422	1,502
Savannah	219	204	234
North East	467	406	526
Upper east	1,242	1,189	1,293
Upper West	375	347	403
**Total**	**41,746**	**41,488**	**41,932**

### Estimation of the roamer population using three source capture-recapture

The estimated size of roamer FSW population in Ghana derived from the three-source capture technique (first capture, second capture and the survey) was 55, 686 (95% CI: 47,686–63,686), (Table 2). As with the previous method, Greater Accra had the highest roamer population 19,081 (95% CI: 14,008–27,421). This was followed by the Western region with 11,535 (95% CI: 6,680–23,331). The region with the lowest roamer population was Western North with an estimate of 118 (95% CI; 73–259).

**Table 2:** Population size estimate of FSW using 3 source capture-recapture for roamers by region in Ghana, 2020.

Region	Female Pop (15–49)	Est	95% LCI	95% UCI	% of Female Pop
Western	571,929	11,535	6,680	23,331	2.02
Central	637,629	4,061	2,553	7,725	0.64
Greater Accra	1,487,470	19,081	14,008	27,421	1.28
Volta	459,072	986	603	2,046	0.21
Eastern	811,662	4,753	2,852	9,178	0.59
Ashanti	1,559,605	5,561	3,784	8,997	0.36
Western North	245,102	118	73	259	0.05
Ahafo	153,596	947	296	14,892	0.62
Bono	293,073	3,488	1,929	8,160	1.19
Bono East	284,288	725	402	1,998	0.26
Oti	182,359	504	365	789	0.28
Northern	463,527	1,345	743	3,363	0.29
Savannah	141,398	208	138	380	0.15
North East	140,030	364	193	941	0.26
Upper East	300,157	1,407	642	5,174	0.47
Upper West	204,319	603	299	2,119	0.30
**Total**	**7,935,216**	**55,686**	**47,686**	**63,686**	**0.70**

### Estimation of the seater and roamer populations using the service multiplier

There were no service data for some of the regions, such as Ahafo, Oti, Savannah, Upper East, Upper West and Western North, as no services were being provided in these regions for FSWs. As expected, regions with no HIV-related FSW interventions contributed zero estimate to the national figure, (Table 3). The total estimated number of both seaters and roamers was 41,153 (95% CI: 37,242–45,984). The highest estimated number of 14,676 (95%% CI: 13,525–16,043) was from Greater Accra, followed by Eastern region 7,701 (95% CI: 6,123–10,375), (Table 3).

**Table 3:** Population size of roamer FSW using service multiplier by region in Ghana, 2020.

Region	Est	95% LCI	95% UCI
Western	7,532	6,567	8,831
Central	1,118	1,039	1,212
Greater Accra	14,676	13,525	16,043
Volta	1,475	1,292	1,717
Eastern	7,701	6,123	10,375
Ashanti	5,082	4,763	5,446
Western North	-	-	-
Ahafo	-	-	-
Bono	1,646	1,525	1,788
Bono East	551	520	586
Oti	-	-	-
Northern	577	535	626
Savannah	-	-	-
North East	-	-	-
Upper east	-	-	-
Upper West	-	-	-
**Total**	41,153	37,242	45,984

### Estimation of the seater population using census

A total of 4,363 seaters were enumerated in all the seater venues across all the sixteen regions of Ghana. The region with the highest seater population is the Greater Accra Region with 1,443 representing 30%, followed by the Ashanti region with 882 representing 20.2%, (Table 4).

**Table 4:** Summary total estimates of seater (census) and roamer (service multiplier, CRC, and 3SCRC) FSW by region according to method.

		Roamers Size Estimation			Seater Size Estimation	All		
		Service Multiplier	Capture–Recapture	3-Source Capture—Recapture	Enumeration of Seaters	Final FSW Size Est	Weighted Frequency Comparison	% of FSWs
Region	Female Pop	Number	Number	Number	Number	Number	Number	%
Western	571,929	7,532	4,128	11,535	869	12,404	5,627	2.17
Central	637,629	1,118	4,602	4,061	132	4,193	5,713	0.66
Greater Accra	1,487,470	14,676	13,751	19,081	1,443	20,524	15,743	1.38
Volta	459,072	1,475	1,045	986	154	1,140	1,450	0.25
Eastern	811,662	7,701	3,426	4,753	110	4,863	5,978	0.60
Ashanti	1,559,605	5,082	6,016	5,561	882	6,443	9,940	0.41
Western North	245,102	-	254	118	44	162	342	0.07
Ahafo	153,596	-	703	947	30	977	1,934	0.64
Bono	293,073	1,646	2,353	3,488	94	3,582	4,495	1.22
Bono East	284,288	551	737	725	156	881	1,365	0.31
Oti	182,359	-	966	504	20	524	2,135	0.29
Northern	463,527	577	1,463	1,345	146	1,491	1,577	0.32
Savannah	141,398	-	219	208	93	301	363	0.21
North East	140,030	-	467	364	10	374	352	0.27
Upper East	300,157	-	1,242	1,407	122	1,529	1,693	0.51
Upper West	204,319	-	375	603	58	661	600	0.32
**Total**	**7,935,216**	**41,153**	**41,746**	**55,686**	**4,363**	**60,049**	**59,307**	**0.76**

* Final estimates is a sum of the 3-source capture-recapture estimate and the seater estimate. (-) No FSW estimates due to absence of programmatic data.

### Overall comparison of estimates according to the method used

Presented in Table 4 are the size estimates of the female sex workers in Ghana using the three population size estimation and the census methods. These include the Census and Enumeration method (seaters), the Capture-Recapture (roamers), Service Multiplier (both seaters and roamers), and 3SCRC (roamers) as well as the weighted frequency estimates based on sampling probability.

The 3SCRC method produced a higher size estimate for the roamer population with 55,686. When the 3SCRC estimate is added to the enumerated seater population of 4,363, the estimated total number of FSWs in Ghana stands at 60,049. This estimate of 60,049 is extremely close to the weighted frequency estimate of 59,307 based on sampling probabilities. The estimated size of FSWs represents about 0.76% of the estimated adult female aged 15–49 years in Ghana. The estimated percentages of FSWs in the regions varies from 0.07% in Western North region to about 2.2% in Western region as shown in Table 4. In Table 5, we provide a brief summary of strengths and limitations for each of the methods used in this study.

**Table 5:** Strengths and limitations of the methods used to estimate the FSW size.

Study Method	Strengths	Limitations
Census	It’s accurate, reliable and robust.	It’s time consuming and resource demanding.
		More suited for counts at the local level.
	It’s appropriate for heterogeneous population within the same geographical location	Not suitable at areas that are geographically diverse and where population at risk are scattered.
Capture-Recapture	This method is much more straightforward to use.	A sufficiently high overlap fraction is required to produce a reliable estimate of the missing population.
	It takes less time and requires less resources to be executed	It requires independence of the sources of data, which is often violated
		Probability of being in a capture is not affected by being/not being in the other capture
Service Multiplier	This method is much more straightforward to use	It depends on high-quality existing data that ensures that each individual member of the high-risk population has a chance of being included. Therefore any program data not well collected will affect its estimates.
	It is perhaps the most commonly used of all the population size estimation methods. They are simple and user-friendly	
		In areas where NGOs are supported by different funding agencies, individuals may be exposed to multiple programmes, but may not be able to differentiate between them and may be reported more than once in programme data.
		If the existing data are poorly documented or are duplicated, the size estimate will be biased.
		The catchment area for the services or institutions must be clear, and should ideally be the same as that covered in the sub-population survey from which multipliers are derived
3-Source Capture Recapture	The 3-source log linear model allows interactions to be examined first and then added to the model as an interaction term which corrects for the dependencies in CRC	It can be used to correct for interdependency between 2 sources but not if there is dependency between 3 sources
	Three interdependencies assumed to be negligible	
	Perfect record-linkage–hierarchic combination of three names to minimize overmatching	

## Discussion

This study used three population size estimation and the census/enumeration methods for FSW in Ghana. The size estimation methods used were service multiplier, capture re-capture (CRC), and three-source capture re-capture (3SCRC). The current size estimation exercise improves upon the two previous size estimation exercises, which used two estimation methods (CRC and service multiplier) including census/enumeration for seaters [18]. In this current study, the final determination of the size estimation was based on census/ enumeration for seaters and the 3SCRC for roamers population. The 3SCRC was agreed upon among stakeholders based on its robustness as reported in the research literature. The 3SCRC method’s strength, includes the perfect record-linkage–hierarchic combination of three names that minimizes overmatching as well as the addition of an interaction term in the model which corrects for the dependencies in CRC. This estimation results of FSWs will provide critical information for macro-and micro-level planning for HIV/STIs care, treatment and AIDS services at the national and regional levels. This will also enable proper resource allocation, program coverage, geographic targeting, and planning of outreach work at all levels.

The total number of seaters nationally was 4,363. The estimated overall total number of FSW in Ghana was 60,049 (a combination of the census figures for seaters and the 3SCRC approach for roamers). This total FSW population estimates is 0.757% (0.751%, 0.763%) of all estimated adult females aged 15–49 years (7,935,216) in Ghana (GSS, 2020).

The 2020 estimate of the size of the overall estimated female sex worker population (55,656) is between the 2015 estimate (65,053) and the 2011 service multiplier estimate (51,937). The differences in the overall estimate for 2020 and that of 2015 and 2011 may arise from methodological approaches in terms of sampling design and statistical estimation method. While the 2015 and 2011 BBS used service multipliers for both seaters and roamers estimation, the 2020 BBS used census for seaters and three-source capture re-capture for roamers. In terms of sampling design and approach, unlike the 2015 BBS, the 2020 study employed a different approach for capture re-capture by randomly sampling 15% of national and regional venues and then extrapolating to obtain the national estimates. The 15% plus extrapolation strategy used in 2020 had operational as well as methodological advantages. It resulted in more stable regional estimates because the number of recaptures was higher and there were many fewer locations with no re-captures. The three-source capture re-capture used both the capture re-capture as well as time-location-sampling (TLS) during the survey to arrive at the size estimates for the roamers. This methodological approach (3-SCRC) differ from those that were employed in Mozambique (sequential sampling, unique object, unique event), Sri Lanka (unique object multiplier and a modified Delphi method), Uganda (Bayesian nonparametric latent-class model), Papua New Guinea (unique object multiplier successive sampling-population size estimation) and Cameroon (Poisson regression model) [10–14].

Field work for the 2020 survey took advantage of lessons learned from previous surveys, reached more sub-district areas, and better documented participation. For example, in 2011 when services were offered in all of the 10 regions, the study covered 10 regional capitals and mapped about 536 venues [19]. In 2015, 783 venues were mapped in 10 regions of Ghana [2]. However, in 2020, the survey covered each of the newly recognized 16 regions of Ghana and mapped 2,482 venues. The overwhelming increase of over a 100% in the number of active venues over that of the 2015 BBS may be partly due to a comprehensive stakeholders’ engagement and also the team’s resolve to reach out to every town, city and village to ensure that no venue was left out. Given that services for FSWs were not being provided in all 16 new regions (previously 10 regions), the service multiplier method which had been the standard for 2011 and 2015 could not be relied upon because it underestimates the FSW population. Additionally, the service multiplier method used in the 2015, highly overestimated the population for Western Region. For the first time in BBS in Ghana, there was a strong congruence of the different estimation methods (CRC, multiplier and 3SCRC) that also aligned with the weighted frequencies based on the survey sampling design.

In this study, it was observed that both the capture re-capture and that of the service multiplier yielded estimates that were for the most part lower than the estimate obtained through 3SCRC in Ghana. In the case of the service multiplier, we observed that 7 out of the 16 regions had no program data. One of the limitations of the service multiplier method is its dependency on programs which mostly result in either underestimation or overestimation of KP population [5]. Also, capture re-capture generally underestimates sizes of KPs [5, 20–22]. Unlike the capture re-capture method, the three-source capture re-capture does not require that the samples collected be independent due to the interaction effect, which controls for the dependency assumption [15, 16, 23]. As a result, we obtained the population size estimates of the roamer FSWs using the 3SCRC method.

### Programmatic implications

There is strong evidence that more than half of FSW surveyed were new entrants and had been practicing sex work for less than five years. Reaching this target group of new entrants to ensure they access HIV prevention services is critical to reducing the risk of infection.

## Conclusion and recommendation

Understanding and knowing your epidemic is fundamental towards developing targeted interventions. We report of PSE for FSWs in 16 regions in Ghana. These estimates are the results of 3SCRC, a robust method currently available for PSE. The female sex worker PSE included in this study should be used guide programmers and policy makers to assess whether HIV care and treatment and other needs are being met and to determine how much and where programs and interventions should focus in the future.

For the first time in BBS in Ghana, there was a strong congruence of the different estimation methods (CRC, multiplier and 3SCRC). The 3SCRC addresses challenges associated with CRC including dependency, closed population and equal capture, making it robust.

### Limitations

The service multiplier in this study had limitations. A number of regions that had no FSW programs did not contribute to the overall size estimates. The service multiplier figure was obviously underestimated. It is possible that some sex workers were shy and may have provided responses which were socially desirable. The spectrum of responses shows that this may have had a limited or no effect. FSW were provided with a compensation of 50 Ghana cedis after the interview. Some sex workers tried to enroll more than once but were identified by the peer educators, research assistants or supervisors. Intimate partners of FSW: Some intimate partners of FSW were unavailable on the day of interview and were unable to meet the research team the following day for their interview.

## Supporting information

S1 Data (ZIP)
